# Post-training breakdown: acute effects of different training types on body hydration status and performance

**DOI:** 10.3389/fpsyg.2024.1528840

**Published:** 2025-01-08

**Authors:** Erkan Demirkan, Mehmet İsmail Tosun, Abdurrahim Kaplan, Mert Ayrancı, Damian George Cosmin, Mustafa Arıcı, Mehmet Kutlu, Veysi Aslan, Michael Favre

**Affiliations:** ^1^Department of Movement and Training Sciences, Faculty of Sport Sciences, Hitit University, Çorum, Türkiye; ^2^Department of Physical Education and Sports, Faculty of Sport Sciences, Hitit University, Çorum, Türkiye; ^3^Department of Recreation, Faculty of Sport Sciences, Hitit University, Çorum, Türkiye; ^4^Department of Physical Education and Sports, Faculty of Physical Education and Sport, Ovidius University of Constanta, Constanta, Romania; ^5^Department of Coaching Education, Graduate School of Health Sciences, Ege University, İzmir, Türkiye; ^6^Intercollegiate Athletics, University of Michigan, Ann Arbor, MI, United States

**Keywords:** athletic training, weight loss, body hydration, wrestlers, performance

## Abstract

**Objective:**

Wrestling is a complex sport that requires a combination of strength, endurance, and wrestling-specific technical training. Endurance activities, such as running, are commonly performed for rapid weight reduction before competition. However, these activities can severely disrupt recovery and lead to significant declines in performance. This study aimed to examine the acute effects of endurance, strength, and wrestling training sessions on hydration status and performance parameters in young wrestlers, providing insights to guide more effective hydration and recovery strategies.

**Method:**

A total of 14 experienced young wrestlers participated in a crossover design, completing endurance, strength, and wrestling training sessions on separate days. The hydration status was assessed through urine specific gravity (USG) before and after each session. The performance measures included hand grip strength, back and leg strength tests, anaerobic power (vertical and horizontal jumps), respiratory muscle strength (maximal inspiratory pressure, MIP), and reaction time assessments.

**Results:**

The endurance training caused the most significant acute reductions in the hydration (USG increase: 0.016 ± 0.005 g/cm^3^, *p* < 0.05) and body mass (BM; −1.89 ± 0.4%, *p* < 0.05), along with immediate performance decrements in the back strength (−7.02 ± 1.2%, *p* < 0.001), right-hand grip strength (−8.79 ± 2.1%, *p* < 0.001), jump height (−7.26 ± 1.8%, *p* < 0.001), and MIP (−9.01 ± 2.3%, *p* < 0.001). The hydration levels in the endurance group did not fully recover by the next day (USG post-training vs. before the next day’s training: *p* < 0.05). In contrast, the strength training improved the reaction time before the next day’s session (+5.6 ± 1.3%, *p* < 0.05), while the attention remained unaffected across all training types.

**Conclusion:**

Endurance training—commonly used for rapid weight loss—can acutely compromise hydration and reduce key performance measures, with recovery taking more time compared to strength or wrestling sessions. Given these findings, endurance sessions should be strategically scheduled before rest days or low-intensity technical training to minimize their negative effects on subsequent performance. Implementing enhanced hydration strategies during endurance-focused sessions may also help mitigate these acute impacts and optimize recovery and performance in young wrestlers.

## 1 Introduction

Wrestling is a weight-category sport that places significant demands on both anaerobic and aerobic energy systems due to its fast-changing movement dynamics and high-intensity physical contact during bouts. Wrestlers must develop not only their physical and physiological conditioning but also their technical, tactical, and psychological skills to succeed (Mirzaei et al., 2009; Demirkan et al., 2015; Schultz et al., 2024). Therefore, wrestling requires key performance components, including anaerobic power, strength, agility, and speed, which are essential for executing complex maneuvers effectively. To optimize these components, the wrestling training season, particularly the general preparatory period, incorporates various training models, such as aerobic endurance, strength, and sport-specific technical training, to achieve peak physical, physiological, and mental performance (Cieśliński et al., 2021).

Dehydration is a major concern in combat sports such as wrestling, where rapid weight loss is a common practice to compete in lower weight categories. Methods such as fluid restriction, prolonged fasting, and high-intensity exercises are frequently used by athletes, increasing the risk of dehydration (Kondo et al., 2018; Ranisavljev et al., 2022). Previous studies have shown that dehydration exceeding 2% of body mass (BM) can impair strength, endurance, reaction time, and overall athletic performance while also compromising heat dissipation and cardiovascular function (American College of Sports Medicine et al., 2007; Cheuvront et al., 2014; Sommerfield et al., 2016; Chycki et al., 2017; Lakicevic et al., 2021). If hydration is not properly monitored, athletes may experience fatigue, heat exhaustion, cramps, and heat stroke (Utter et al., 2012). Rapid weight loss also negatively impacts both physical health and mental wellbeing, particularly when performed over short durations (Yarar, 2022). Each training type selected in this study has unique physiological and practical demands that justify their inclusion. Endurance training plays a dual role in wrestling preparation: it develops the aerobic capacity needed for prolonged matches and serves as a common strategy for rapid weight loss before competitions (Ghaemi et al., 2014). Wrestlers often rely on high-intensity endurance exercises to reduce body mass quickly, which increases the risk of dehydration and its associated performance decrements (Lambert and Jones, 2010). In contrast, strength training focuses on enhancing muscular strength and power—key components for executing explosive techniques during bouts (Sangirov and Aripova, 2020). Finally, wrestling-specific training combines the physical, technical, and tactical requirements of the sport, ensuring that both energy systems are simultaneously engaged under sport-specific conditions. Moreover, understanding the recovery duration following each type of training can provide coaches with valuable insights for optimizing training schedules. By identifying which training type causes greater dehydration and performance decrements, coaches can better determine the appropriate sequencing of endurance, strength, and wrestling-specific training. This information will help ensure that recovery periods are adequately planned and that subsequent training sessions are not negatively impacted by fatigue or inadequate hydration. Numerous studies have examined rapid weight loss methods used by athletes (Drid et al., 2021; Roklicer et al., 2022; Ranisavljev et al., 2022; Castor-Praga et al., 2021) and investigated the effects of dehydration on athletic performance and physiological parameters (Sommerfield et al., 2016; Utter et al., 2012; Barley et al., 2020), no study has directly compared the acute effects of endurance, strength, and wrestling-specific training on hydration status and performance. Addressing this gap is crucial for optimizing hydration strategies and training programs for wrestlers.

The purpose of this study was to compare the acute effects of endurance, strength, and wrestling-specific training performed on different days on body hydration (BH) status and performance parameters. In addition, the study aimed to evaluate the recovery of hydration and performance levels immediately before the subsequent training session. It was hypothesized that endurance training would result in greater body weight loss and hydration disturbances compared to strength and wrestling training, leading to more pronounced decrements in performance parameters. However, this does not imply that endurance training should be eliminated; rather, its inclusion in wrestling training programs should be carefully planned, with appropriate hydration strategies to minimize its adverse effects.

## 2 Materials and methods

### 2.1 Participant

Before beginning the study, the sample size was calculated using G Power 3.1.9.7 (Dusseldorf, Germany), which concluded that 12 participants were sufficient to achieve a power of 95% (*β* = 0.95), an effect size (f) of 0.534, and an error rate of *α* = 0.05. To account for potential dropouts, 14 young male wrestlers with at least 4 years of wrestling experience were recruited (age: 15.07 ± 0.73; height: 167.14 ± 9.88 cm; body mass: 61.15 ± 12.64 kg; BMI: 21.58 ± 2.5; body fat: 15.19 ± 3.22%; fat-free mass: 51.55 ± 9.56 kg). The inclusion criteria were as follows: (1) male wrestlers aged between 14 and 16 years; (2) ranked in the top five in their weight category at the most recent national championship; (3) a minimum of 4 years of regular wrestling training experience; and (4) no health problems that could prevent participation. Wrestlers were excluded if they had a history of smoking, chest diseases, or sports injuries within the last 12 months. All participants stayed together in a sports complex, followed the same meal plan, and trained under the guidance of the same coach 6 days a week. Written informed consent forms, approved by the Ethics Committee of Non-Invasive Research, Hitit University (Decision Number: 2024–07), were obtained from all participants and their parents/guardians. The study adhered to the Declaration of Helsinki. Design and Procedures.

The study used a repeated-measures crossover design. Individuals who volunteered to join the study and met the inclusion criteria completed the necessary paperwork (Informed Consent Form – Informed Parental/Guardian Consent Form). After receiving approval from the principal investigator, the training protocols and tests outlined in the study were initiated. All participants were given a comprehensive introduction to the tests 12 and 6 days before the start of the study, and trial measurements were conducted. Three distinct training protocols (wrestling training, strength training, and endurance training) were administered sequentially, with a one-week interval between each protocol. All sessions were held on the same day and at the same time each week (Tuesdays at 3:00 pm): the wrestling training on 8th October, the strength training on 15^th^ October, and the endurance training on 22nd October. The participants were encouraged to perform at their peak during both the training and test protocols. Before and after each training protocol, as well as before the training on the following day, the participants’ body hydration levels were measured using the urine specific gravity (USG) method, and the body fat percentages were assessed by performing bioelectrical impedance analysis. The back and leg strength were evaluated with a back and leg dynamometer, while the hand grip strength was measured using a hand grip dynamometer. The respiratory muscle strength was measured based on the maximal inspiratory pressure (MIP) test. In addition, the participants’ performances were analyzed using vertical and horizontal jump tests, as well as attention and reaction tests. Before the training protocols, the athletes refrained from any exercise for 24 h and from consuming food for at least 3 h. They were instructed to avoid acidic or diuretic foods and beverages for 48 h before each protocol. All tests were carried out by researchers who specialized in these measurements.

In our study, three different training protocols were applied. The training protocols were as follows:

**Wrestling Training (WT)**: The participants, under the supervision of their coach, completed a warm-up and stretching protocol specific to wrestling before beginning the exercises aimed at enhancing their wrestling experiences. The wrestlers engaged in matches against opponents within their own weight categories, with each match lasting 3 min, followed by a 2-min rest period. After each rest period, the partners were rotated and new matches commenced. Each wrestler completed a total of eight matches. During the matches, the coach provided feedback as needed, pausing the bout to ensure correct techniques were applied. A 4-min rest period was implemented after every two matches. After all matches were concluded, a 15- min cool-down protocol was performed, with the entire training session lasting approximately 90 min.

**Circuit Strength Training (ST)**: Before starting the workout, the participants completed a 10-min warm-up run and dynamic stretching exercises. Following this preparatory phase, the participants performed the strength training in a well-ventilated standard indoor fitness facility using a circuit method. The specifics of the strength training protocol are detailed below (Table 1).

**Table 1:** Exercise and set-rest structure of the circuit strength training protocol.

Targeted region	Exercise name	Repetition count	Set count	Rest duration (seconds)
Legs	Bulgarian Split Squat (Dumbbells)	8	3	60
	Box Jump	10	3	60
Back	Bent Over Row	8	3	60
	Lat Pulldown	10	3	60
Chest	Dumbbell Bench Press	10	3	60
	Push-Up	10	3	60
Arms	Dumbbell Curl and Shoulder Press	10	3	60
	Triceps Pushdown	12	3	60
Core	Russian Twist (with Weights)	20	3	60
	Plank with Arm Lift	1 min.	3	60

**Endurance Training (ET)**: Before starting the endurance workout, the participants completed a 10-min warm-up run and dynamic stretching exercises. Following this preparation, they undertook a continuous running protocol covering a distance of 10 km on a standard 400-meter rubber-surfaced athletics track. The running intensity was set to target 70–75% of each participant’s maximal heart rate, calculated using the Karvonen formula. The heart rates were monitored in real time throughout the run using an optical heart rate sensor (Polar Verity Sense) to ensure that the participants maintained the target intensity range of 70–75% of the maximal heart rate during the session.

### 2.2 Test protocol

#### 2.2.1 Measurement of the inspiratory muscle strength

To assess the strength of the inspiratory muscles during the Muller maneuver, the MIP test was employed, a validated method recognized for its ability to gage respiratory muscle strength (American Thoracic Society/European Respiratory Society, 2002; Volianitis et al., 2001). This evaluation utilized a portable device (Micro Medical-Carefusion Micro RPM, United Kingdom) (Stavrou et al., 2021), which provided real-time measurement readings. During the testing, the participants securely held the device with both hands and sealed their lips tightly around the flanged mouthpiece. The procedure required the participants to exhale fully to reach the residual volume, followed by performing a maximal inspiratory effort sustained for over 1 s (Stavrou et al., 2021; American Thoracic Society/European Respiratory Society, 2002). A nose clip was applied to prevent nasal air leakage, and all measurements were conducted in a standing position (Lomax et al., 2015). Each participant completed three MIP trials per timepoint, with the highest value recorded for subsequent statistical analysis.

#### 2.2.2 Urine specific gravity measurement

Urine specific gravity measurement was conducted using a refractometer (Atago, Japan) to assess the participants’ hydration status. Before the measurement, the refractometer was calibrated with distilled water in accordance with the manufacturer’s instructions. Fresh urine samples were collected from the participants in sterile containers, and the refractometer’s measuring tip was directly immersed in the urine sample to record the USG values. The measurements were performed at room temperature under stable lighting conditions, providing a reliable indicator of individual hydration status (Demirkan et al., 2024).

#### 2.2.3 Back and leg strength measurement

Back and leg strength measurements were conducted using a Takei mechanical dynamometer (Takei Scientific Instruments Co., Japan) to evaluate the participants’ muscular strength. For the back strength assessment, the participants stood with their feet shoulder-width apart on the device’s platform, maintaining a straight back with slightly bent knees, and grasped the dynamometer’s handlebar. They were instructed to pull upward with maximum effort. For the leg strength assessment, the participants assumed a deeper knee-bent position to increase leg muscle activation, pulling with maximum effort once again. Three trials were performed for each measurement, and the highest recorded strength value was documented (Eyuboglu et al., 2019).

#### 2.2.4 Hand grip strength measurement

Hand grip strength measurement was performed using a digital hand dynamometer (Takei Scientific Instruments Co., Japan) to evaluate the participants’ hand and forearm muscle strength. During the measurement, the participants stood upright with their arms relaxed at their sides. Holding the dynamometer with their dominant hand, they were instructed to apply maximum force. Three trials were conducted for each participant, and the highest recorded force value was documented. Throughout the measurement, the participant’s wrist was maintained in a neutral position without any support from the body (Patti et al., 2022).

#### 2.2.5 Vertical and horizontal jump test

Vertical jump force was measured using a JumpMat device (Takei TKK 5406) to assess the participants’ lower extremity explosive power. The participants jumped with maximum effort while keeping their hands on their hips, and the device automatically calculated the jump height based on the airtime. For the horizontal jump assessment, the participants were made to perform a maximal forward jump to evaluate their lower extremity strength and explosive power, with the jump distance measured from the starting point. Three trials were conducted for both measurements, and the highest recorded value was documented (Kotsifaki et al., 2023).

#### 2.2.6 Attention and reaction test

In this study, a web-based interactive device providing multisensory stimuli (CatchPad - Smart Training Platform, 2024) was used to assess the attention and reaction. The athletes participated in a 60-s attention test using six touch pads, each placed 20 cm apart. During the test, the athletes were instructed to extinguish a randomly activated red light, which appeared among six different colors, as quickly as possible. Correct and incorrect responses were recorded, along with the measurement of the minimum, maximum, and average reaction times.

### 2.3 Statistical analysis

All statistical analyses were performed using IBM SPSS Statistics 26.0 (IBM Corp., Armonk, NY, United States), and graphical representations were generated with GraphPad Prism 10.0 (GraphPad Software, San Diego, CA, United States). Data normality was assessed using the Shapiro–Wilk test, confirming a normal distribution. Sphericity was evaluated using Mauchly’s test, and the Greenhouse–Geisser correction was applied when the sphericity assumptions were violated. Changes across the time points were analyzed with one-way repeated measures ANOVA. Within-group comparisons (pre-test, post-test, and before the next day’s training measurements) were conducted using Tukey’s post-hoc test, while between-group comparisons were performed with Bonferroni-adjusted pairwise analyses to minimize errors due to multiple testing. Statistical significance was set at a *p*-value of <0.05. The results were reported as mean ± standard deviation (SD), with confidence intervals provided by the post-hoc tests to further detail the significance levels.

## 3 Results

Table 2 presents the changes in the body mass and hydration status across the three time points (pre-training, post-training, and before the next day’s training) for each training protocol. The repeated measures ANOVA revealed statistically significant differences in the body mass between the time points for all training protocols (ST: *F* = 45.32, η^2^ = 0.28; WT: *F* = 39.45, η^2^ = 0.26; ET: *F* = 51.73, η^2^ = 0.32; *p* < 0.05). Regarding the hydration status, significant differences were observed in the pre-training and post-training values for the WT (*F* = 21.84, η^2^ = 0.19; *p* < 0.05) and ET (*F* = 27.12, η^2^ = 0.21; *p* < 0.05) protocols. In addition, significant decreases in the hydration status were found between the post-training and before the next day’s training values for the ST (*F* = 18.35, η^2^ = 0.17; *p* < 0.05) and ET (*F* = 24.56, η^2^ = 0.20; *p* < 0.05; Table 2).

**Table 2:** Changes in the body mass and hydration status according to each training protocol at the three time points.

V	TP	Pre-training	% Dif	dif	Post-training	% Dif	dif	Pre-training*	f	*Post hoc*	*p*
BM	ST	^1^61.15 ± 12.64	−1.39	^♣^−0.85	^2^60.30 ± 12.4	1.54	0.93	^3^61.23 ± 12.55	330.68	1–2	0.001
										2–3	0.001
	WT	^1^60.11 ± 12.55	−0.96	^♦^0.58	^2^59.53 ± 12.5	0.90	1.12	^3^60.66 ± 12.49	323.05	1–2	0.001
										2–3	0.002
	ET	^1^61.12 ± 12.51	−1.89	^♠^1.17	^2^59.96 ± 12.41	1.60	0.96	^3^60.92 ± 12.69	327.693	1–2	0.001
										2–3	0.001
*p*				♦-♠ 0.001	*p*		NS				
BH (Usg)	ST	^1^1.023 ± 0.003	−0.29	^♣^0.003	^2^1.026 ± 0.006	−0.38	^♣^−0.004	^3^1.022 ± 0.002	1113234.65	2–3	0.029
	WT	^1^1.025 ± 0.003	0.48	^♦^0.005	^2^1.030 ± 0.003	0.58	^♦^0.006	^3^1.036 ± 0.047	59446.367	1–2	0.012
	ET	^1^1.022 ± 0.005	1.56	^♠^0.016	^2^1.038 ± 0.016	−1.44	^♠^0.015	^3^1.023 ± 0.005	286281.689	1–2	0.005
										2–3	0.018
*p*				♣-♠ 0.004 ♦-♠ 0.018	*p*		♣-♠ 0.001 ♦-♠ 0.022				

*p* < 0.001; V, variable; BM, body mass; BH, body hydration; TP, training protocol; ST, strength training; WT, wrestling training; ET, endurance training; *next day training; NS, no significant. ♣, strength training; ♦, wrestling training; ♠, endurance training 1, pre-training; 2, post training; 3, next day training.

**Figure 1:**
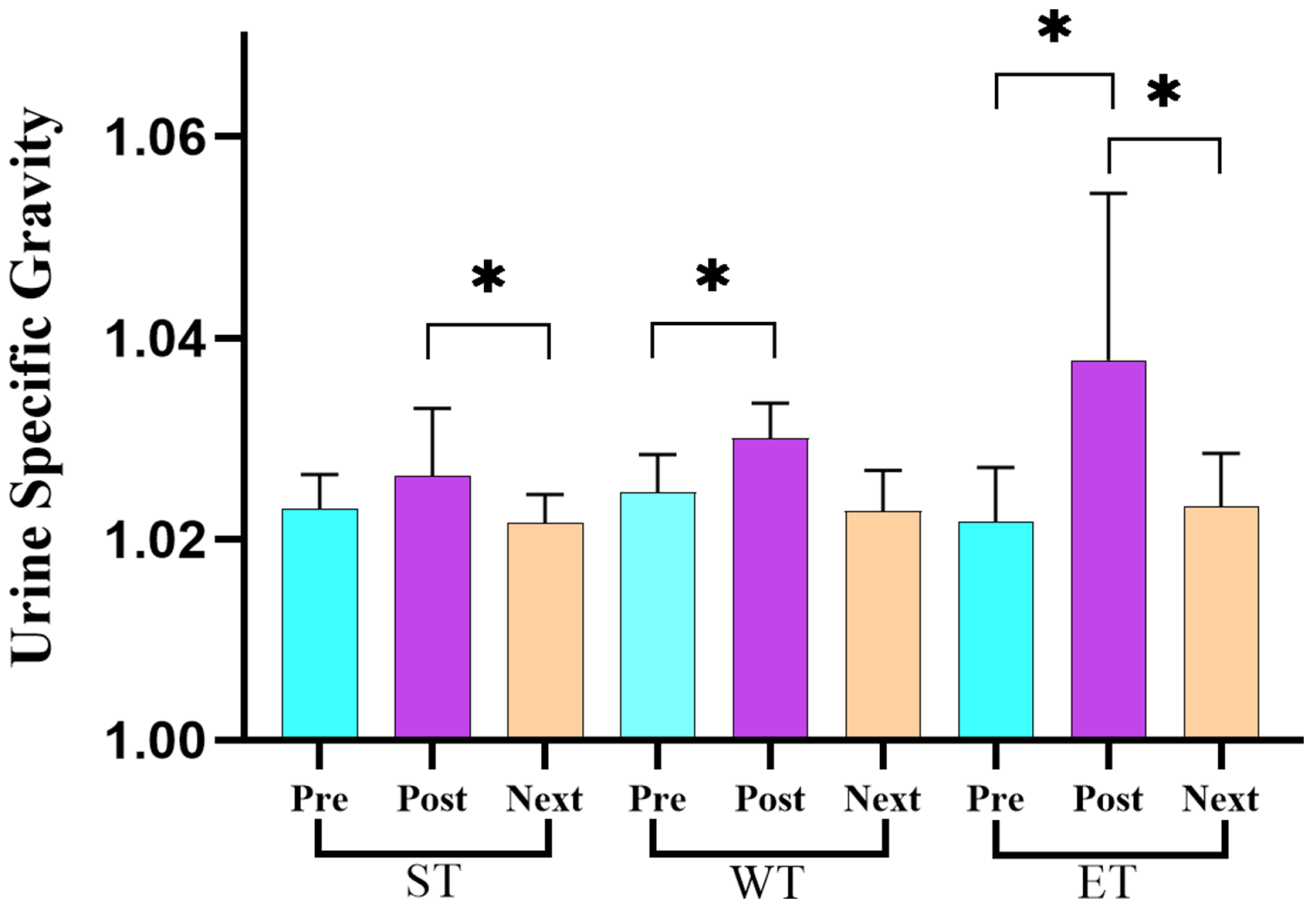
Changes in the urine specific gravity across the three training protocols at the pre-training, post-training, and before the next day’s training time points.

Table 2 presents the changes in the body mass (BM) and body hydration (BH) status across the three measurement points (pre-training, post-training, and before the next day’s training) for each training protocol (ST, WT, and ET). The repeated measures ANOVA revealed statistically significant differences in the body mass between the pre- and post-training values for all training protocols (ST: *F* = 42.15, η^2^ = 0.29; WT: *F* = 38.67, η^2^ = 0.27; ET: *F* = 49.23, η^2^ = 0.32; *p* < 0.05). Similarly, significant differences in the body mass were observed between the post-training and before the next day’s training values for all training protocols (*p* < 0.05). For the hydration status, significant differences were found in the pre- and post-training values for the WT (*F* = 19.42, η^2^ = 0.18; *p* < 0.05) and ET (*F* = 24.58, η^2^ = 0.21; *p* < 0.05; Figure 1) protocols. In addition, significant decreases in the hydration status were detected between the post-training and before the next day’s training values for the ST (*F* = 17.62, η^2^ = 0.16; *p* < 0.05) and ET (*F* = 22.41, η^2^ = 0.20; *p* < 0.05) protocols. When comparing the BM and BH losses and gains among the groups, significant differences in the body mass change were observed between the WT and ET models (*p* < 0.05). However, no statistically significant changes in the body mass were detected between the post-training and before the next day’s training values within the groups (*p* > 0.05). For the hydration status, the ET protocol showed statistically significant differences in both pre- and post-training and before the next day’s training values compared to the ST and WT protocols (*F* = 23.67, η^2^ = 0.22; *p* < 0.05; Table 2).

**Figure 2:**
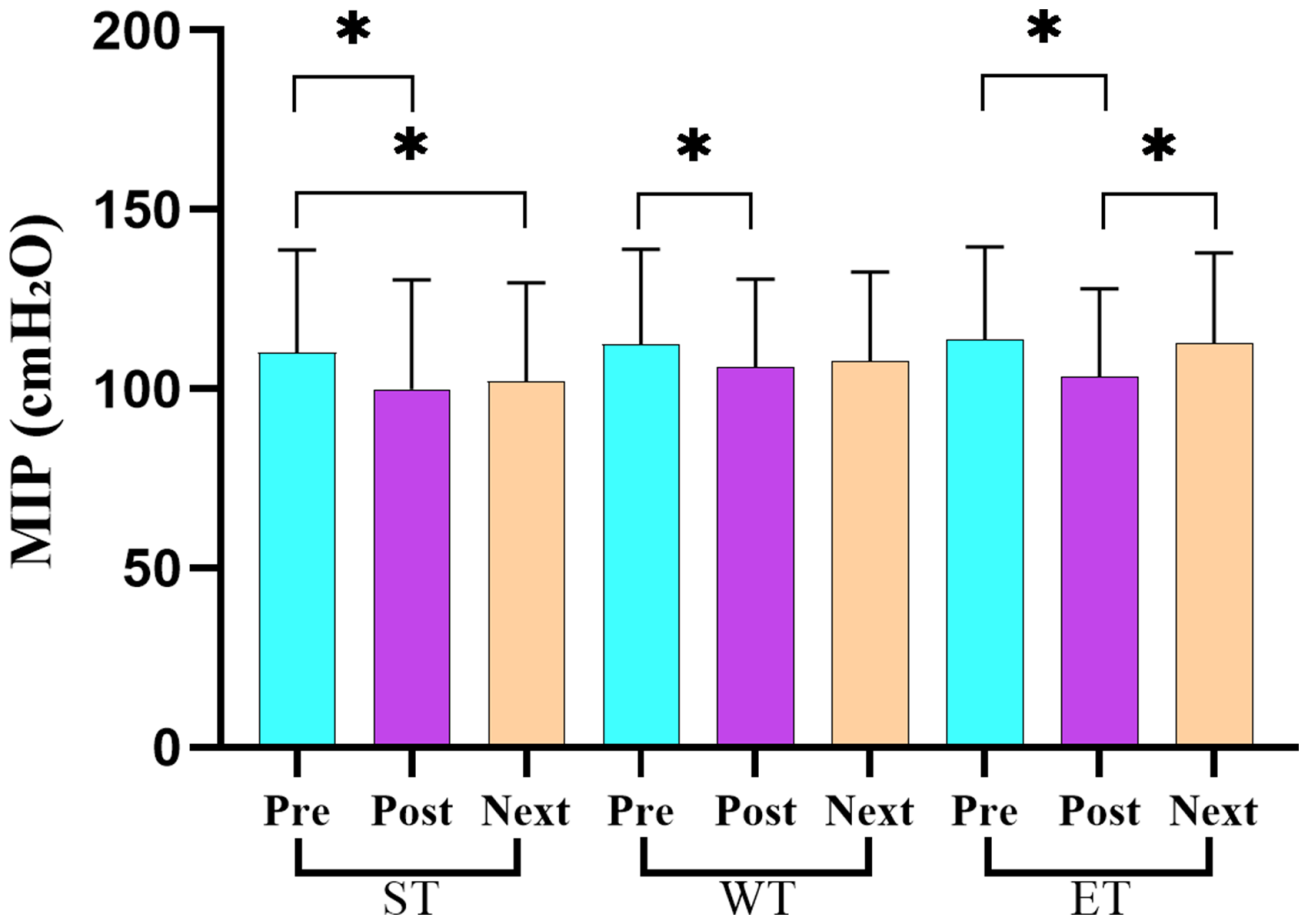
Maximal inspiratory pressure (MIP) changes across the three training protocols at the pre-training, post-training, and before the next day’s training time points.

Table 3 presents the average scores and standard deviations (mean ± SD) for the strength and power variables across the three time points (pre-training, post-training, and before the next day’s training) for each training protocol. The repeated measures ANOVA revealed significant losses in the performance for the endurance training in the leg strength, vertical jump, horizontal jump, and maximal inspiratory pressure (MIP) between the pre- and post-training measurements (leg strength: *F* = 42.15, η^2^ = 0.28; vertical jump: *F* = 38.21, η^2^ = 0.24; horizontal jump: *F* = 49.12, η^2^ = 0.32; MIP: *F* = 45.32, η^2^ = 0.30; *p* < 0.001). In addition, significant decreases in the horizontal jump and MIP performances were observed for the strength training (horizontal jump: *F* = 21.67, η^2^ = 0.18; MIP: *F* = 19.42, η^2^ = 0.17; *p* < 0.05) and wrestling training (horizontal jump: *F* = 18.34, η^2^ = 0.16; MIP: *F* = 17.68, η^2^ = 0.15; *p* < 0.05; Figure 2) protocols. The Tukey’s post-hoc analysis further confirmed these differences, particularly highlighting the pronounced decrements in the ET protocol when compared to the ST and WT protocols (*p* < 0.05).

**Table 3:** Changes in the strength and power variables according to each training protocol at the three time points.

V	TP	Pre-training	% Dif	dif	Post-training	% Dif	dif	Pre-training*	f	post	*p*
Back strength (kg)	ST	^1^118.78 ± 27.04	−4.05	^♣^−4.82	^2^113.96 ± 27.77	3.63	^♣^4.14	^3^118.10 ± 26.18	265.15	1–2	0.001
	WT	115.64 ± 27.47	−4.04	^♦^−4.68	110.96 ± 26.74	2.6	^♦^2.86	113.82 ± 28.05	252.87	-	NS
	ET	^1^116.71 ± 29.8	−7,02	^♠^−8.20	^2^108.51 ± 27.45	5.23	^♠^5.68	^3^114.19 ± 26.49	232.99	1–2	0.002
										2–3	0.017
*p*				NS		*p*	NS				
Leg strength (kg)	ST	^1^142.39 ± 31.94	−9.55	^♣^−13.61	^2^128.78 ± 28.81	2.30	^♣^2.97	^3^131.75 ± 35.08	252.21	1–2	0.001
										1–3	0.001
	WT	^1^129.89 ± 26.62	−3.02	^♦^−13	^2^116.89 ± 23.71	2.55	^♦^9.04	^3^125.93 ± 25.86	356.020	1–2	0.001
										2–3	0.030
	ET	^1^122.69 ± 24.62	−8,19	^♠^−10.05	^2^112.64 ± 25.2	5.82	^♠^6.56	^3^119.20 ± 24.6	332.88	1–2	0.001
*p*				NS		*p*	NS				
Right hand grip strength (kg)	ST	^1^36.25 ± 8.79	−5.40	^♣^−1.96	^2^34.29 ± 8.78	1.69	^♣^0.59	^3^34.87 ± 7.60	264.20	1–2	0.009
	WT	^1^37.18 ± 8.69	−5.97	^♦^−2.22	^2^34.96 ± 8.08	6.95	^♦^2.43	^3^37.39 ± 8.64	266.34	1–2	0.001
										2–3	0.015
	ET	^1^36.25 ± 8.36	−8.79	^♠^−3.19	^2^33.06 ± 8.13	12.76	^♠^4.22	^3^37.28 ± 8.78	258.01	1–2	0.003
										2–3	0.004
*p*				NS	*p*		NS				
Left hand grip strength (kg)	ST	^1^34.07 ± 8.59	6.41	^♣^2.19	36.26 ± 8.85	−5.26	^♣^−1.91	34.35 ± 8.12	256.44	1–2	0.046
	WT	35.26 ± 7.61	−0.38	^♦^−0.14	35.13 ± 9.44	4.47	^♦^1.57	36.70 ± 9.42	241.96	–	NS
	ET	35.02 ± 2.30	2.02	^♠^0.71	35.73 ± 2.37	−3.66	^♠^−1.31	34.42 ± 2.18	248.11	–	NS
*p*				NS	*p*		NS				
Vertical jump (cm)	ST	43.00 ± 8.12	−2.81	^♣^−1.21	41.79 ± 6.63	2.36	^♣^1.0	42.78 ± 6.61	515.35	–	NS
	WT	45.07 ± 9.26	−2.52	^♦^−1.14	43.93 ± 9.19	0.15	^♦^0.07	44.00 ± 7.17	399,528	–	NS
	ET	^1^42.75 ± 8.78	−7.26	^♠^−3.11	^2^39.64 ± 8.62	4.86	^♠^1.13	^3^41.57 ± 8.89	388.22	1–2	0,001
										1–3	0,043
										2–3	0,001
*p*				NS	*p*		NS				
Horizontal jump (cm)	ST	^1^216.21 ± 24.42	−2.34	^♣^−5.07	^2^211.14 ± 23.6	2.57	^♣^5.43	^3^216.57 ± 26.61	1067.29	1–2	0,002
										2–3	0,049
	WT	^1^216.79 ± 22.26	−4.61	^♦^−10	^2^206.79 ± 22.47	3.45	^♦^7.14	^3^213.93 ± 21.06	1426.61	1–2	0,032
	ET	^1^210.96 ± 7.74	−6.53	^♠^−13.78	^2^197.18 ± 8.09	4.52	^♠^8.93	^3^206.11 ± 7.86	1568.60	1–2	0,001
										2–3	0,002
*p*				♣-♠ 0.033	*p*		NS				
MIP (cmH_2_O)	ST	^1^110.143 ± 28.61	−9.27	^♣^−10.21	^2^99.929 ± 30.43	2.21	^♣^2.21	^3^102.143 ± 27.44	184.855	1–2	0,001
										1–3	0,002
	WT	^1^112.429 ± 26.5	−5.59	^♦^−6.29	^2^106.143 ± 24.49	1.48	^♦^1.27	^3^107.714 ± 24.84	264.381	1–2	0,001
	ET	^1^113.714 ± 25.83	−9.01	^♠^−10.25	^2^103.464 ± 24.48	8.90	^♠^9.21	^3^112.679 ± 25.15	274.565	1–2	0,001
										2–3	0,002
*p*				NS	*p*		♣-♠ 0.015 ♦-♠ 0.008				

*p* < 0.001; V, variable; BM, body mass; MIP, maximal inspiratory pressure; TP, training protocol; ST, strength training; WT, wrestling training; ET, endurance training; *next day training; NS, no significant; cmH2O, Unit of pressure measurement; cm, centimeter; kg, kilogram. ♣, strength training; ♦, wrestling training; ♠, endurance training 1, pre-training; 2, post training; 3, next day training.

**Figure 3:**
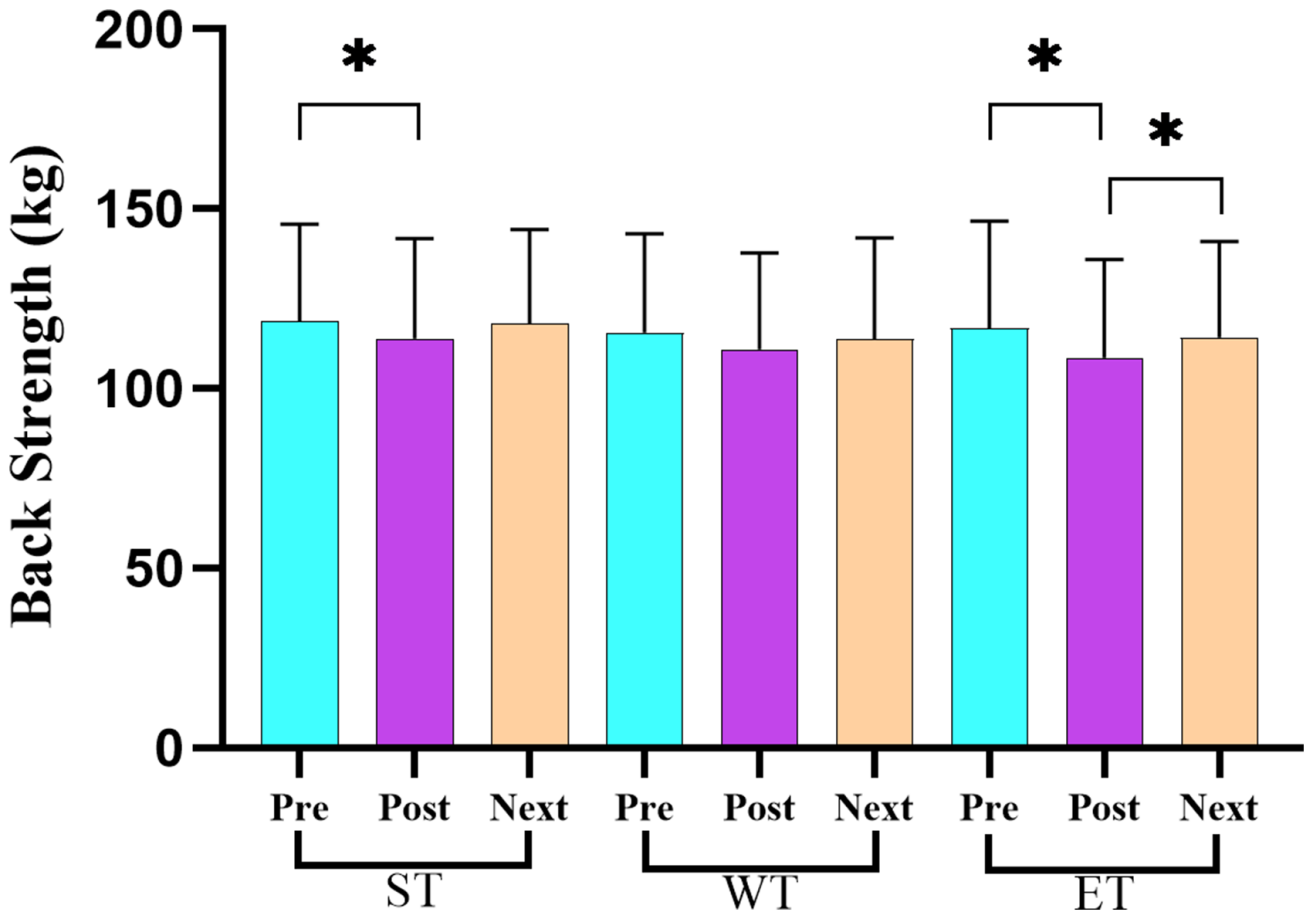
Changes in the back strength across the three training protocols at the pre-training, post-training, and before the next day’s training time points.

**Figure 4:**
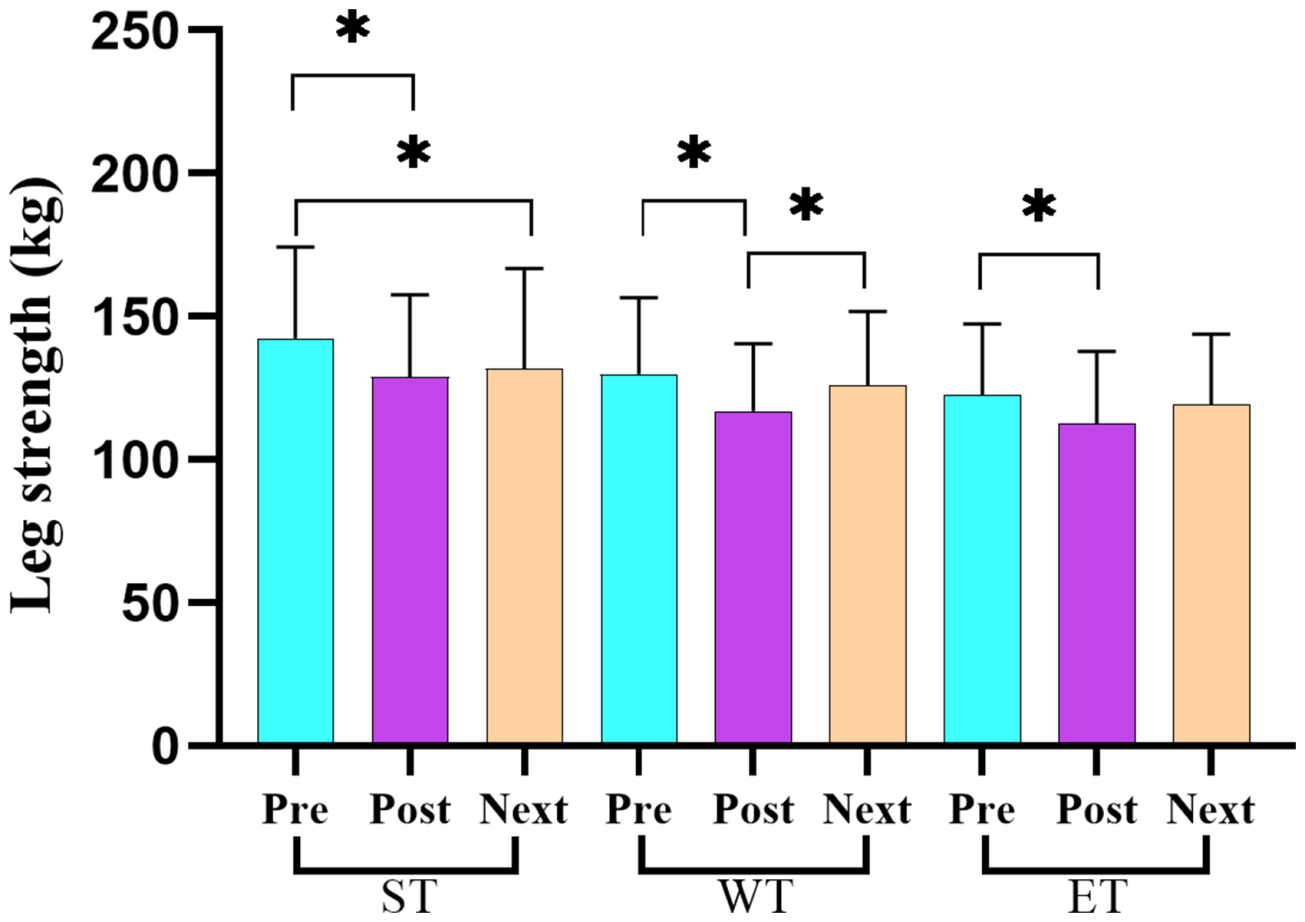
Changes in the leg strength across the three training protocols at the pre-training, post-training, and before the next day’s training time points.

**Figure 5:**
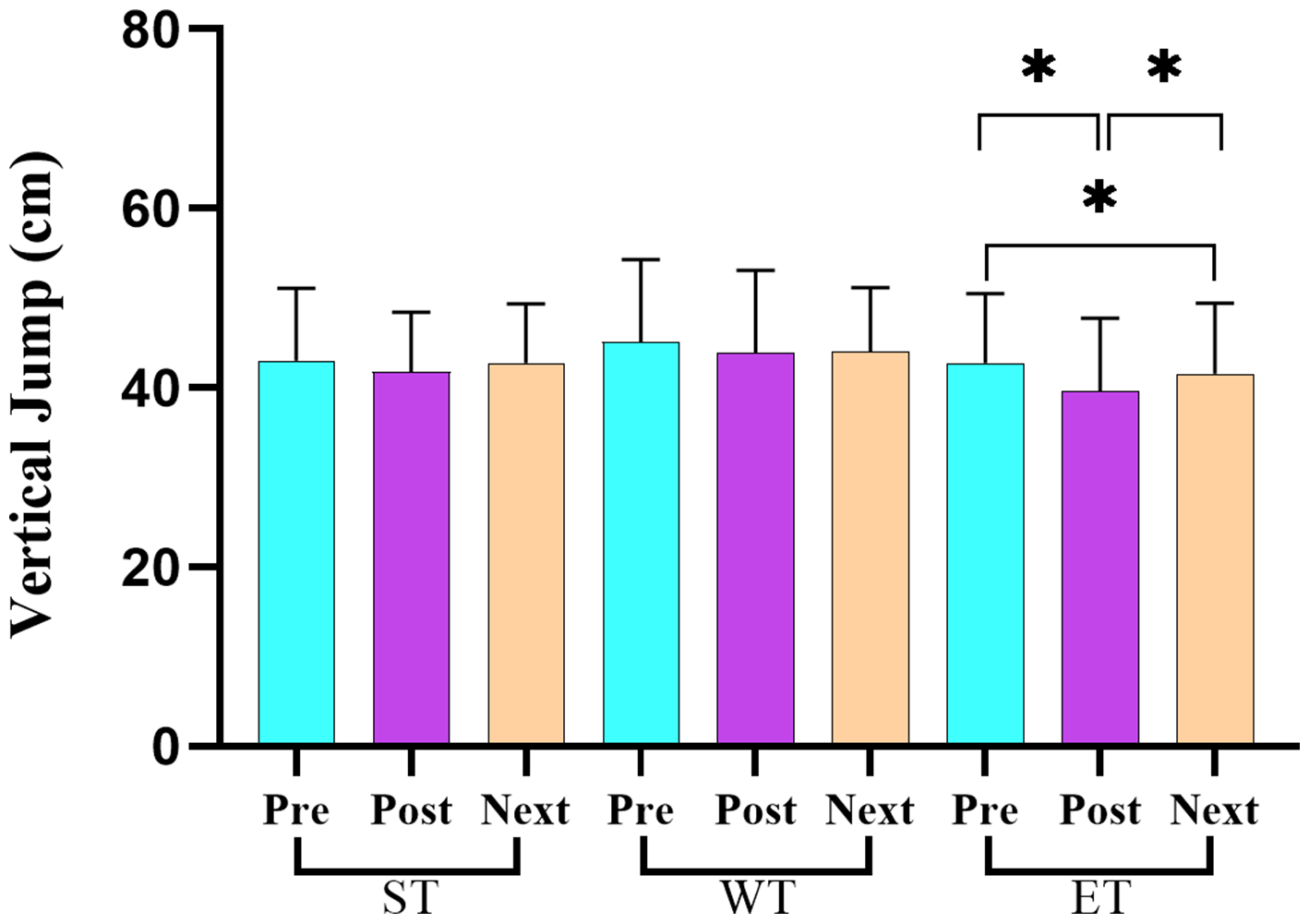
Changes in the vertical jump performance across the three training protocols at the pre-training, post-training, and before the next day’s training time points.

**Figure 6:**
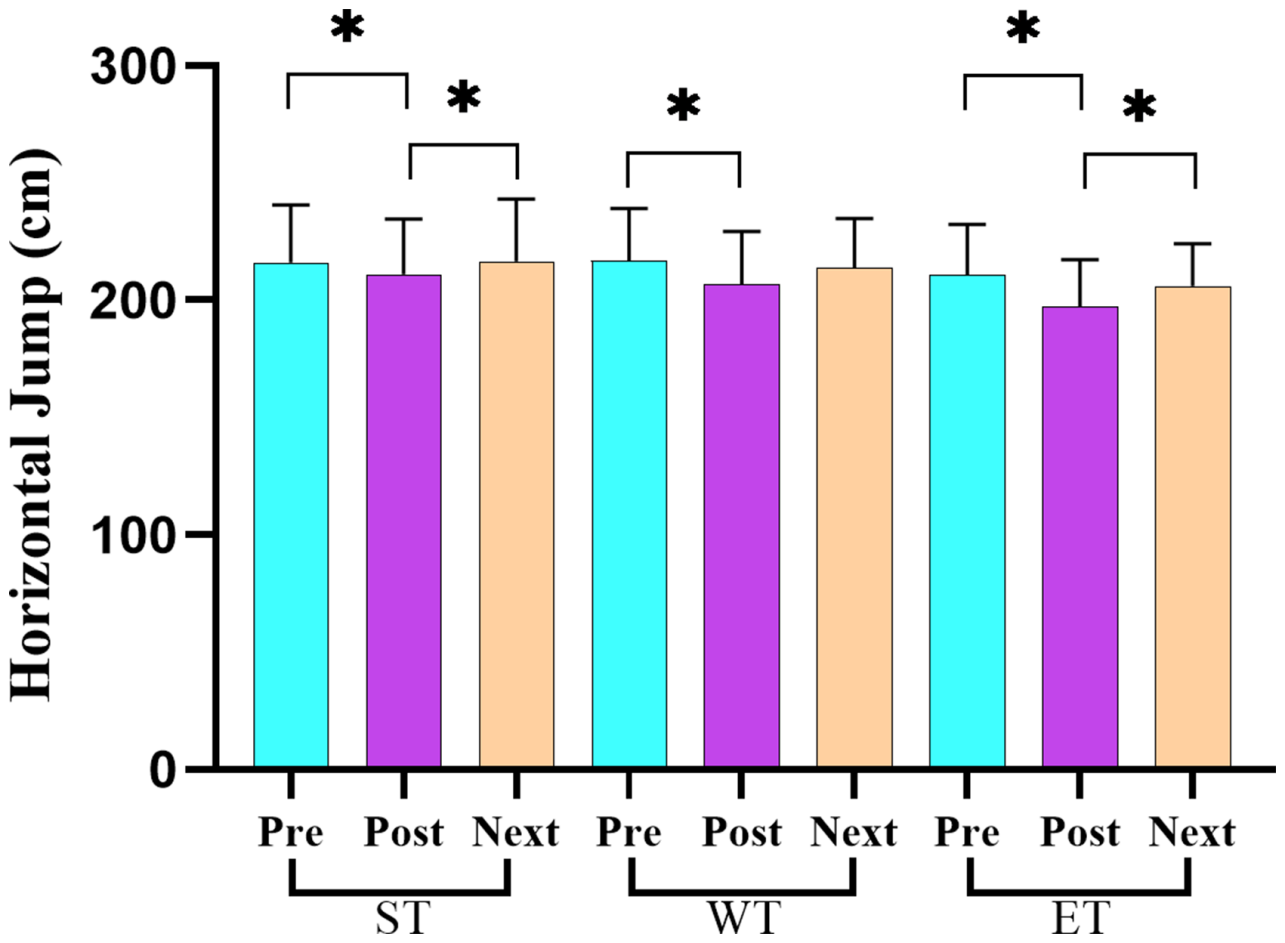
Changes in the horizontal jump performance across the three training protocols at the pre-training, post-training, and before the next day’s training time points.

Table 3 presents the average scores and standard deviations (mean ± SD) for the strength and power variables across the three measurement points (pre-training, post-training, and before the next day’s training) for each training protocol (ST, WT, and ET). The repeated measures ANOVA revealed significant losses in the back strength performance between the pre- and post-training values for both ST (*F* = 28.73, η^2^ = 0.24; *p* < 0.05) and ET (*F* = 33.62, η^2^ = 0.27; *p* < 0.05) protocols. However, a significant recovery in the back strength performance was observed in the ET protocol between the post-training and before the next day’s training measurement points (*F* = 15.32, η^2^ = 0.18; *p* < 0.05; Figure 3). For the leg strength performance, significant decreases were observed between the pre- and post-training values in all training models (ST, WT, and ET; *F* = 40.21, η^2^ = 0.30; *p* < 0.05; Figure 4). In the hand grip strength performance, significant losses were observed in the right-hand grip strength between the pre- and post-training for all training models (*F* = 22.14, η^2^ = 0.19; *p* < 0.05). In addition, a significant recovery in the right-hand grip strength performance was observed between the post-training and before the next day’s training time points for the WT (*F* = 18.56, η^2^ = 0.17; *p* < 0.05) and ET (*F* = 21.78, η^2^ = 0.20; *p* < 0.05) protocols but not for the ST protocol. For the vertical jump performance, significant decreases were observed in the ET protocol between the pre- and post-training measurement points (*F* = 26.45, η^2^ = 0.23; *p* < 0.05), followed by a significant increase between the post-training and before the next day’s training measurement points (*F* = 14.61, η^2^ = 0.16; *p* < 0.05; Figure 5). For the horizontal jump performance, significant decreases occurred in all three training models between the pre- and post-training measurement points (*F* = 30.54, η^2^ = 0.25; *p* < 0.05). However, significant recoveries were observed in the ST (*F* = 16.48, η^2^ = 0.18; *p* < 0.05) and ET (*F* = 19.27, η^2^ = 0.20; *p* < 0.05) protocols between the post-training and before the next day’s training measurement points (Figure 6). The losses in the horizontal jump performance were significantly greater in the ET protocol compared to the ST protocol (*p* < 0.05). For the maximal inspiratory pressure, significant decreases were found between the pre- and post-training values for all training models (*F* = 25.12, η^2^ = 0.22; *p* < 0.05). However, the MIP showed a significant recovery in the ET protocol between the post-training and before the next day’s training values (*F* = 17.83, η^2^ = 0.19; *p* < 0.05). Furthermore, the recovery of the MIP in the ET protocol was significantly higher compared to the other training protocols (*p* < 0.05).

**Figure 7:**
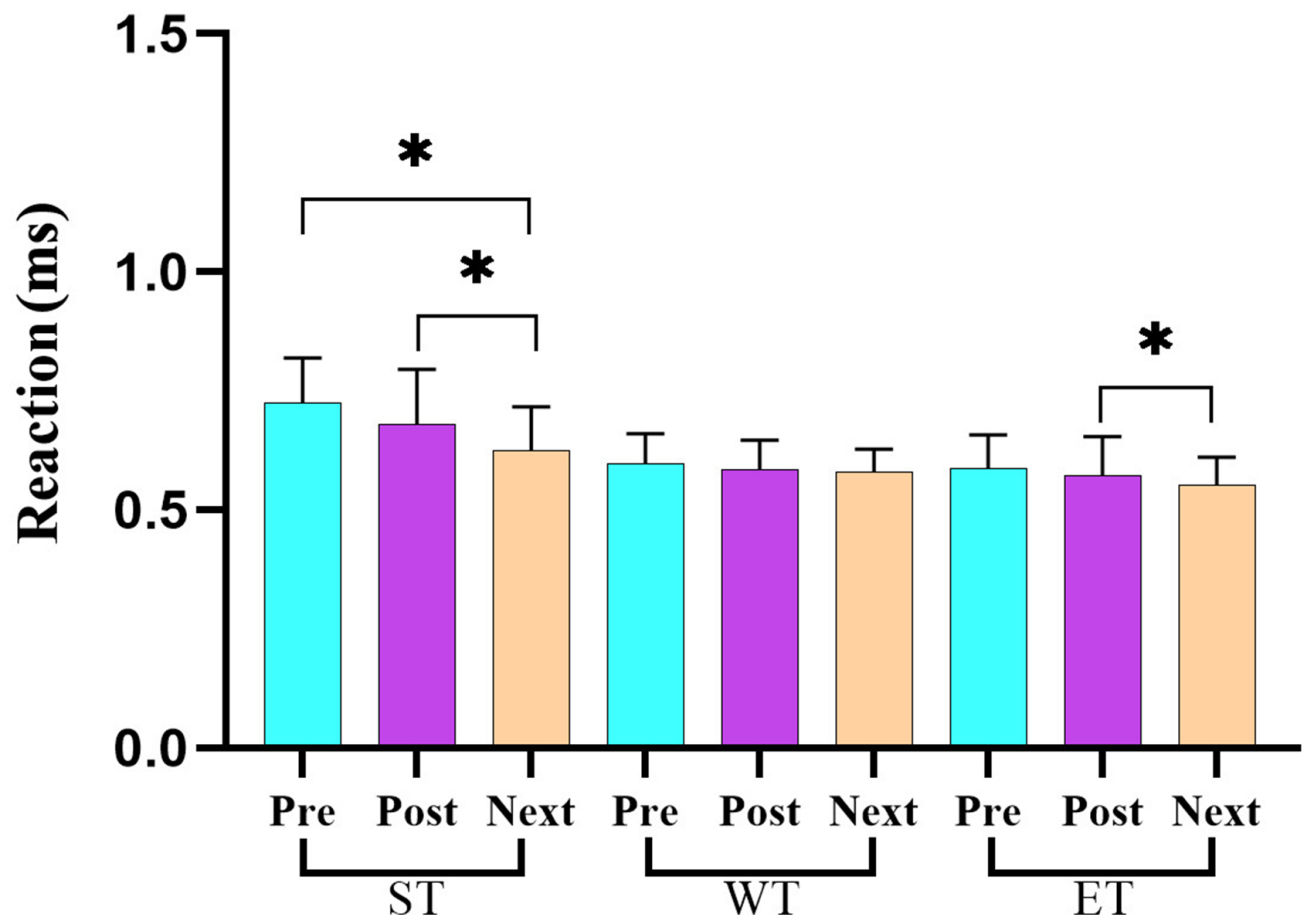
Reaction time changes across the three training protocols at the pre-training, post-training, and before the next day’s training time points.

Table 4 presents the mental attention and reaction time variables across the three measurement points (pre-training, post-training, and before the next day’s training) for each training protocol (ST, WT, ET). For the mental attention variable, the repeated measures ANOVA showed no statistically significant differences within the groups or between the groups across all time points (*p* > 0.05). For the reaction time, significant changes were observed within certain protocols. In the ST protocol, the reaction times significantly decreased before the next day’s training compared to the pre-training (*F* = 14.27, η^2^ = 0.18; *p* = 0.001) and post-training (*F* = 9.45, η^2^ = 0.15; *p* = 0.027) values. Similarly, in the ET protocol, a significant decrease in the reaction time was observed between the pre-training and before the next day’s training values (*F* = 12.56, η^2^ = 0.17; *p* = 0.004; Figure 7). When comparing the differences in the reaction times among the training protocols, a significant difference was found between the ST and WT protocols (*p* = 0.037; Table 4). This indicated that while the reaction times improved notably in the ST group, no such changes were observed in the WT protocol.

**Table 4:** Changes in the attention and reaction variables according to each training protocol at the three time points.

V	TP	Pre-training	% Dif	dif	Post-training	% Dif	Dif.	Pre-training*	f	*Post hoc.*	*p*
Attention	ST	61.571 ± 8.71	1.50	^♣^0.93	62.500 ± 11.37	6.4	^♣^4	66.500 ± 9.12	800.465	1–2,1-3,2–3	NS
	WT	69.357 ± 5.00	0.30	^♦^0.21	69.571 ± 6.36	3.28	^♦^2.29	71.857 ± 6.02	3533.702	1–2,1-3,2–3	NS
	ET	72.179 ± 7.3	2.91	^♠^2.21	74.286 ± 9.09	0.33	^♠^0.25	74.536 ± 7.2	1434.993	1–2,1-3,2–3	NS
*p*				NS	*p*		NS				
Reaction (ms)	ST	^1^0.726 ± 0.09	−6.33	^♣^−0.046	^2^0.680 ± 0.11	−7.94	^♣^0.054	^3^0.626 ± 0.09	783.208	1–3	0.001
										2–3	0.027
	WT	0.598 ± 0.062	−2.34	^♦^−0.014	0.584 ± 0.062	−0.51	^♦^0.003	0.581 ± 0.046	1813.860	1–2,1-3,2–3	N.S
	ET	^1^0.588 ± 0.069	−2.72	^♠^−0.016	^2^0.572 ± 0.083	−3.32	^♠^0.019	^3^0.553 ± 0.057	987.170	1–3	0.004
*p*				NS	*p*		♣-♦ 0.037				

*p* < 0.001; V, variable; TP, training protocol; ST, strength training; WT, wrestling training; ET, endurance training; *next day training; NS, no significant; ms, millisecond. ♣, strength training; ♦, wrestling training; ♠, endurance training 1, pre-training; 2, post training; 3, next day training.

## 4 Discussion

The objective of this study was to evaluate changes in body hydration status and performance across three different training protocols. This is the first study to investigate changes in hydration status and performance based on different training models in wrestlers. In this study, the endurance training model was confirmed to be the type of training model in which body weight and body hydration level changes are most prevalent compared to strength and wrestling training models, as we hypothesized. The primary findings of the study indicated that the athletes who were in a dehydrated state at all the time points (pre-training, post-training, and before the next day’s training) had USG values higher than 1.020 g/cm^3^ (in the range of 1.022–1.038 g/cm^3^). The National Collegiate Athletic Association (NCAA) has identified a USG value of less than or equal to 1.020 g/cm^3^ as an indicator of proper hydration (Borden et al., 2019). Barley et al. (2020) reported that a USG value greater than 1.020 is typically considered hypohydrated. Based on these findings, it is obvious that all the wrestlers were in a dehydrated state throughout all the training sessions. Our study findings concerning these aspects indicate that the wrestlers were consistently in a dehydrated position as a consequence of a lack of education. Bialowas et al. (2023) reported that judo athletes started competition in a dehydrated condition and remained dehydrated at the end of the event. In our study, when investigating the findings related to the body mass and body hydration status during the training sessions and before the next day’s training, the most prominent results were observed in the ET model. In the ET model, the losses in the body mass [1.17 kg (1.89%)] and hydration status [0.016 g/cm^3^ (1.56%)] between the pre- and post-training time points were greater than in the ST and WT models [in ET: BM 1.17 kg (1.89%), BH 0.016 g/cm^3^ (1.56%); in ST: BM 0.85 kg (1.39%), BH 0.003 g/cm^3^ (0.29%); in WT: BM 0.58 kg (0.96%), BH 0.005 g/cm^3^ (0.48%)]. In addition, these findings indicate that the USG increased concurrently with the body mass losses. A few studies investigating the hydration status of athletes have shown findings similar to those of our study. A study conducted by Ceylan et al. (2022) confirmed that 5% dehydration could result in an increase in USG and a decrease in body mass. Furthermore, of interest from these findings was that although the body mass values returned to the pretest levels after the post-training and before the next day’s training, the fact that the athletes’ body hydration status was still not back to the proper hydration levels during the post-training and before the next day’s training indicated that the wrestlers were constantly mildly to moderately dehydrated. Another study conducted by Demirkan et al. (2014) stated that despite a 1.4 kg weight gain occurring over the 14-h period between the post-weigh-in and the competition, no significant change in body hydration status was observed (1.028 g/cm^3^ to 1.027 g/cm^3^, respectively). Meanwhile, Oppliger et al. (1996) reported that fluid homeostasis could be renewed within 24 to 48 h. Ceylan et al. (2023) confirmed that although male judo athletes’ body mass significantly increased by 4.2% from the weigh-in to 24-h post-competition, USG values were still higher a week before the competition and during the official weigh-in compared to the 24-h post-competition values. In view of the abovementioned literature and our findings, these results suggest that athletes are not able to fully recover body hydration status, despite gaining body weight. Similarly, Connor et al. (2022) stated that after a 5.3% loss of body mass, followed by a 24- to 26-h recovery period of proper refueling and rehydration strategies, no impairment in blood markers and performance indices was found. Based on our study findings, we suggest that ET may lead to greater body weight loss and increased dehydration compared to the other training regimes. Therefore, coaches and athletes should be mindful of maintaining athletes’ hydration levels, especially during endurance training, and athletes should be provided time to intake fluids during training sessions.

When considering the performance parameters, the main findings indicated that the ET model caused a higher loss of performance in the back strength (7.02%), right-hand grip strength (8.79%), vertical and horizontal jumps (7.26, 6.53% respectively), and MIP (9.01%) compared to the ST and WT models (Table 2). These results are especially concerning because hand grip strength is one of the most important performance components for grappling sports, such as wrestling, as a variety of wrestling moves are used during a match. Our findings reported that the right-hand grip strength decreased significantly in all training models, with the impairment in the ET model being greater than in the other training models. However, there was no decrease in the left-hand grip strength, most likely due to the wrestlers predominantly using the right hand or the depletion of muscle glycogen reserves in the ET model (Kristiansen et al., 2000; Hearris et al., 2018). It may also be that there is a more developed proprioceptive and neural feedback mechanism in the dominant hand (De Honorato et al., 2021). Therefore, it suggests that the symptoms of fatigue or loss of strength in the right hand are more pronounced due to the greater sensitive perception of the nervous system. The present study is the first to demonstrate changes in body hydration and performance decrements based on different training models. In previous studies, the findings have been unclear as some of them have stated no changes in performance due to acute weight loss (Artioli et al., 2010; Connor et al., 2022; Judelson et al., 2007; Yang et al., 2018; Mauricio et al., 2022), while others have indicated a decrease in performance (Ceylan et al., 2022; Barbas et al., 2011; Kraemer et al., 2001; Degoutte et al., 2006). For example, Artioli et al. (2010) reported that after a 5% loss of body mass among judo athletes, followed by a 4-h recovery period, no negative effect on arm power was observed. Connor et al. (2022) indicated that after a rapid weight loss of 5.3%, followed by more than 24 h of recovery, there was no negative impact on performance indices. Judelson et al. (2007) confirmed that a body mass loss of up to 4.8% failed to affect vertical jump height, peak lower-body power, and peak lower-body force; however, it significantly declined the ability to execute a resistance exercise bout. Yang et al. (2018) reported that after a rapid weight loss of 5%, followed by a 16 h recovery period, no deleterious effect on physiological parameters, such as V02max, was observed. Mauricio et al. (2022) reported in a systematic review study that a rapid weight reduction of ≤5% in body mass in less than 7 days did not impair performance outcomes for strength and power measures in combat athletes. McKenna and Gillum (2017) conducted a study on the effects of exercise-induced dehydration on anaerobic power in male collegiate wrestlers. The authors concluded that a reduction of up to 3% in body mass did not result in deleterious effects on lower-body anaerobic power. Contrary to these findings, Ceylan et al. (2022) stated that a 5% reduction in body mass impaired dynamic and isometric strength in the upper limbs, as well as impaired performance in tasks requiring aerobic and anaerobic energy sources in judo-specific performance. Barbas et al. (2011) investigated the physiological and performance responses following a typical weight loss regimen (~ 6%) in elite wrestlers during a simulated one-day tournament of Greco–Roman wrestling. The authors concluded that performance markers such as vertical jump, hip back strength, bear hug strength, and hand grip strength (which declined earlier after match 2) deteriorated by 13–16% after the third bout compared to baseline values. In a similar study, Kraemer et al. (2001) examined the physiological effects of a 2-day freestyle wrestling tournament following a 1-week weight loss period of 6%. They found that lower-body power and upper-body isometric strength significantly declined during the tournament. In another study, Degoutte et al. (2006) reported that left-hand grip performance (53.6 ± 2.7 vs. 50.4 ± 2.5 kg) significantly declined, with a ~ 5% body mass loss in judo athletes. In our study, the findings showed that the body mass loss was lower (in the range of 0.96 kg to 1.89 kg) compared to previous studies. The difference in the rate of body mass loss may explain the contrasting findings on performance parameters in the literature.

In addition to the aforementioned findings, another result from this study showed that all performance parameters returned to the baseline levels by the next day, except for the leg strength (first day: 142.39 ± 8.54 kg, second day: 131.75 ± 9.37 kg) and MIP (first day: 110.14 ± 7.65, second day: 102.14 ± 7.34) parameters in the ST model and the vertical jump parameters (first day: 42.75 ± 2.07, second day: 41.57 ± 2.10) in the ET model (Table 2). These findings indicate that coaches should pay attention to these parameters with regard to recovery, depending on the training models.

Regarding the attention and reaction time variables, no difference was observed in the mental attention between the pre-training, post-training, and before the next day’s training time points (Table 3). The protection of the mental attention throughout the training sessions may most likely be due to the focus on the workout during the training sessions.

However, these findings showed that the ST model led to a greater decrease in the reaction times compared to the ET and WT models. This can be attributed to the skeletal muscles’ increasing ability to generate force due to the learning effect on the central and peripheral nervous systems, as well as motor unit firing, which may result in a decline in muscle reaction speed. Schmidt and Lee (2005) confirmed that repetitions during training create a “motor memory” for the nervous system, making movements faster and more efficient. The authors reported that this learning process allowed athletes to respond faster to a specific motor task. Therefore, reinforcing the memory after training could make it possible to have a faster motor response time the following day. In contrast to these findings, Sant’Ana et al. (2017) investigated reaction time and kick impact in taekwondo athletes.

They reported that the reaction time and kick impact were impaired in the test, which had an intensity equivalent to aerobic power. The authors suggested that the reason for this is that exercise above the ventilatory threshold causes a decrease in cerebral blood flow in specific brain areas involved in responding to peripheral visual stimuli. We suggest that future studies should thoroughly investigate the effects of attention and reaction time related to rapid weight loss in athletes, due to the lack of research on this subject.

## 5 Conclusion

In conclusion, the present study indicated that the body hydration status (0.29–1.56%) and body mass loss (0.96 to 1.89%) changed according to the training models. Furthermore, although the body weight increased before the next day’s training, there was no significant change in the body hydration status. These findings showed that the endurance training, compared to the other training models in our study, was the type of training in which the body weight and body hydration level changes were most prevalent. Therefore, athletes should be encouraged to intake fluids regularly throughout training sessions, especially during endurance training, to optimize body hydration status and performance. Therefore, in particular, immediately before events, endurance training may be limited for wrestlers who are adjusting their body weight due to insufficient recovery of body hydration. Related to these findings, it was observed that the ET model caused greater adverse effects on the back strength, right-hand grip strength, vertical and horizontal jump, and MIP performance compared to the ST and WT models. However, the ST model led to a greater decline in the reaction times compared to the ET and WT models. Regarding the recovery process for the before the next day’s training time point, all performance parameters returned to the baseline levels by the next day, except for the leg strength and MIP performance in the ST model and the vertical jump performance in the ET model.

## 6 Limitations

The study was conducted on male junior wrestlers aged between 14 and 16 years, with at least 5 years of training experience. In addition, the study was executed during the preparatory session and considered only the training models that were prepared by the coaches. It was limited to endurance, strength, and wrestling training models. In the endurance training, all wrestlers performed a 10 km run, and the strength training included the exercises listed in Table 1.

## Data Availability

The raw data supporting the conclusions of this article will be made available by the authors, without undue reservation.
